# Examination of Behavioral and Neuropsychological Characteristics of Hungarian Patients with Juvenile Idiopathic Arthritis: A Cross-Sectional Analysis

**DOI:** 10.3390/children12081057

**Published:** 2025-08-12

**Authors:** Diána Garan, Lilla Lengvári, Andrea Ponyi, Márton Szabados, Gyurgyinka Gergev, Imre Bozi, Wouter Wijker, Tamás Constantin

**Affiliations:** 1 Division of Pediatric Rheumatology and Immunology-Pediatric Center, Semmelweis University, Tűzoltó Street 7-9, 1094 Budapest, Hungary; lilla.lengvari@semmelweis.hu (L.L.); ponyi.andrea@semmelweis.hu (A.P.); szabados.marton@semmelweis.hu (M.S.); gergev.gyurgyinka@gyerekklinika.com (G.G.); constantin.tamas@semmelweis.hu (T.C.); 2 Auxiliis Pharma Budapest Kft., 1037 Budapest, Hungary; imre.bozi@auxiliis-pharma.com (I.B.); wouter.wijker@auxiliis-pharma.com (W.W.)

**Keywords:** juvenile arthritis, psychology, behavior, mood disorders, pediatric rheumatology

## Abstract

Background: Children with juvenile idiopathic arthritis (JIA) may experience chronic pain, contributing to psychological distress. Objective: The objective was to assess neuropsychological functions and behavioral skills in patients with JIA and identify demographic and disease-related factors influencing these outcomes. Methods: This cross-sectional study evaluated 112 patients at the Division of Pediatric Rheumatology, Semmelweis University (2015–2016). Participants completed psychological assessments using the Child Behavior Checklist and Woodcock–Johnson III Tests. Examined variables included demographic (age and sex), clinical (age at diagnosis and disease activity), and treatment-related factors (therapy type and duration). Treatment groups comprised (a) combination therapy with TNF inhibitor and methotrexate (MTX) (n = 60), (b) MTX monotherapy (n = 34), and (c) TNF inhibitor monotherapy (n = 18). Results: Neuropsychological variables showed no clinically significant differences between treatment groups. These skills were unaffected by age, sex, therapy duration, or disease activity. Pathological behavioral scores were significantly higher (*p* < 0.05) in younger patients (<7 years), with females showing greater susceptibility to anxiety and depression (*p* < 0.05). Conclusions: No clinically significant psychological impairments were observed in our cohort. Further research is warranted to clarify the significance of abnormal behavioral scores. Psychological care provision remains vital for improving the quality of life in JIA patients.

## 1. Introduction

Juvenile idiopathic arthritis (JIA) represents one of the most prevalent chronic childhood diseases, with an estimated incidence of 1.6–23 per 100,000 children annually worldwide [1]. Children with JIA may experience chronic pain, reduced mobility, joint stiffness, growth retardation, and frequent medical visits, all of which can disrupt daily functioning and contribute to psychological distress [1,2,3]. Primary therapeutic objectives encompass not only maintaining physical health but also preserving emotional well-being and mental health.

Studies have shown that children and adolescents with JIA face increased risk of psychiatric disorders, anxiety, depression, and behavioral problems compared to healthy peers [4,5,6,7]. These psychological complications can adversely affect disease management, treatment adherence, and overall quality of life.

Previous publications examining the safety and effectiveness of TNF inhibitors (etanercept, infliximab, and adalimumab) have reported psychological or neuropsychological adverse events (AEs) during treatment [8,9,10,11,12,13,14,15]. Lovell et al. (2000) reported personality changes and severe depression during etanercept treatment [8]. Subsequently, various authors observed neuropsychological events (attention deficits, learning difficulties, and working memory impairment), as well as behavioral (anxiety and aggression) and neuropsychiatric changes [8,9]. The incidence of these AEs appeared to be lower or absent with infliximab and adalimumab in other studies [10,11]. However, a detailed analysis found neuropsychological events (AEs) occurred in 12.3% of the study population on infliximab therapy, and similar AEs were found in 23.6% of etanercept patients [12]. A phase IV multicenter registry evaluating the long-term safety of etanercept, methotrexate, and combination therapy reported psychological AEs in 0–2.72% of patients, with anxiety, depression, and neuropathy being most common in the etanercept group [13]. Although these studies documented the occurrence of AEs, they provided limited detailed psychological analysis. Consensus regarding the prevalence and severity of psychological problems among JIA patients across different treatment regimens remains lacking.

Currently, some studies highlight depression as a significant JIA comorbidity. A German study reported a depression prevalence of 9.5%, exceeding the global pediatric average [7]. However, systematic reviews reveal conflicting findings regarding relationships between symptoms of depression and anxiety with disease characteristics in JIA [16].

Some authors suggest that disease activity has less impact on behavioral skills, while functional disability and pain may affect specific cognitive and behavioral abilities [17,18,19,20]. Conversely, a recent study revealed that JIA patients responding poorly to therapy, even with biologic agents, experienced particularly elevated depression scores [7].

Fair et al. identified the lack of comprehensive longitudinal studies examining the effects of treatment on mental health [16], and we agree with this. These gaps highlight the need for research identifying patients most vulnerable to mental health problems and improved strategies for the treatment and prevention of depression and anxiety in young people with JIA.

Understanding behavioral and neuropsychological development in children with chronic diseases, particularly JIA, requires consideration of multiple influencing factors. Environmental, cultural, and genetic factors may lead to different results across psychological studies. There are missing data on the psychological state of Hungarian JIA patients, and our results may help address this knowledge deficit. Our objective was to assess the psychological well-being of patients with JIA, investigate potential neuropsychological and behavioral effects of various therapies, and identify potential influencing factors. We initially planned an informative cross-sectional study with a single psychological assessment to detect significant abnormalities. Considering our findings, we plan to repeat assessments after 10 years and have started a longitudinal follow-up study.

## 2. Materials and Methods

### 2.1. Patients

This cross-sectional study involved 112 JIA patients treated at Semmelweis University’s Pediatric Rheumatology Unit between 2015 and 2016. All assessments were conducted with informed consent from legal guardians.

Inclusion criteria included the following:

• Individuals between 2 and 18 years of age at diagnosis;
• JIA diagnosis according to the ILAR criteria;
• Treatment with TNF inhibitor and/or methotrexate (MTX) and/or sulfasalazine (SSZ).

We excluded patients with SJIA to decrease the clinical heterogeneity of our cohort.

Treatment distribution: 60 patients (53.57%) received combined therapy (TNF inhibitor (TNFi) + (MTX) and/or SSZ), 34 patients (30.35%) received MTX alone, and 18 patients (16.07%) received TNFi monotherapy (etanercept or adalimumab) at the time of psychological assessment.

### 2.2. Main Outcome Variable

The general study population (n = 112) underwent a single psychological evaluation during regular rheumatological follow-up. Patients aged 6 years or older underwent a neuropsychological examination (n = 90).

## 3. Procedures

### 3.1. Measurement of Psychological Function

The Child Behavior Checklist (CBCL) Manual for ages 4–18 (Hungarian adaptation 1996) and Woodcock–Johnson Cognitive Ability Tests were utilized [21]. This second test assessed cognitive abilities in individuals aged 2 to 90 years, offering comprehensive measurement of general intellectual ability, specific cognitive skills, language proficiency, and academic achievement. Parents completed questionnaires addressing general behavior. Teacher and child (for older than 7 years of age) versions of questionnaires exist as well, but we decided to use the parental form for various reasons—for, example we could examine more patients in a wider age range (4–18). Eight psychological variables (behavioral skills) were analyzed: attachment, thinking, concentration, anxiety–depression, somatization, sociability, aggression, and deviant behavior. Score ranges (0–100/per variable) were transformed into percentile ranks with classifications of normal (0–90th percentile), borderline (90.1–97th percentile), and abnormal/pathological (>97th percentile).

### 3.2. Measurement of Neuropsychological Function

Testing examined attention, working memory, and learning using the Woodcock–Johnson Tests of Cognitive Abilities. Score classifications were very superior (≥131), superior (121–130), high average (110–120), average (90–110), low average (80–89), low (70–79), and very low (≤69) [21,22]. For clinical interpretation, we categorized scores as normal (≥90) or abnormal (<90).

### 3.3. Disease-Specific Parameters

At the time of psychological assessment and initial disease activity (JADAS-71-P: Juvenile Arthritis Disease Activity Score-71 at the time of psychological examination, JADAS-71-I: Juvenile Arthritis Disease Activity Score-71 initial disease activity, at the start of the therapy), duration of therapy and demographics (age at diagnosis, age at psychological assessment, and gender) were recorded. JADAS-71 disease activity was defined by JADAS-71 scores for polyarthritis as inactive (≤1.0), low (1.1–3.8), moderate (3.9–10.5), or high (>10.5); for oligoarthritis, categories included inactive (≤1.0), low (1.1–2.0), moderate (2.1–4.2), and high (>4.2) [23]. These clinical variables were stratified, and the treatment groups were compared by aligning them.

### 3.4. Statistical Analysis

Descriptive and exploratory analyses were performed. Between-group comparisons of demographic and psychological parameters were performed using a one-way ANOVA method (followed by Tukey’s post hoc test where appropriate) for continuous variables and chi-square or Fisher’s exact test for categorical variables. A two-way ANOVA was employed for stratified analyses, examining interactions between treatment group and stratification variables. The distribution of normal versus abnormal neuropsychological results and normal, borderline, and abnormal psychological results across strata was evaluated using chi-square and Fisher’s exact tests.

To analyze the impacts of seven factors (gender, age, age at diagnosis, type of therapy, duration of therapy, JADAS-71-P, and JADAS-71-I) on NP skills, main effects and interactions were evaluated using a two-way ANOVA method. Statistical significance was set at *p* < 0.05. To avoid false conclusions, additional requirements included a minimum of 4 patients per stratum and an R-square > 0.3. Analyses were conducted using the SAS 9.4 TS Level 1M3 software package on the X64_8PRO platform.

The procedures adhered to the standards established by the respective local committee. The approval of the local ethics committee was obtained in 2014 (Approval Number: SE TUKEB 124/2014).

## 4. Results

### 4.1. Patient Characteristics and Mean Scores Were Compared Across Three Therapeutic Groups (Table 1 and Table 2)

JIA subtype distribution varied by treatment group: undifferentiated and oligoarthritis predominated in the MTX group, extended oligoarthritis in the TNF inhibitor group, and RF-negative polyarthritis in the combination group. Gender, age at diagnosis, age at psychological assessment, duration of therapy, and JADAS-71-P did not differ significantly between treatment groups. Understandably, the initial disease activity in the combined therapy group was significantly higher than in other groups. NP parameters were within the normal range (attention: MTX—108.78 ± 19.3, TNF inhibitor—108.64 ± 18.45, combined—111.90 ± 17.58; learning: MTX—99.96 ± 10.05, TNF inhibitor—107.29 ± 14.97, combined—105.90 ± 12.92; working memory: MTX—110.85 ± 14.35, TNF inhibitor—110.14 ± 17.73, combined—110.29 ± 12.71), with no significant differences between treatment groups (Table 1). Behavioral parameter analysis revealed no significant differences in proportions of normal, borderline, or pathological values between groups (Table 2). Pathological results were observed only in the combination treatment group, and more borderline cases were detected in the MTX group.

### 4.2. Neuropsychological Parameters Between Treatment Groups

**Figure 1:**
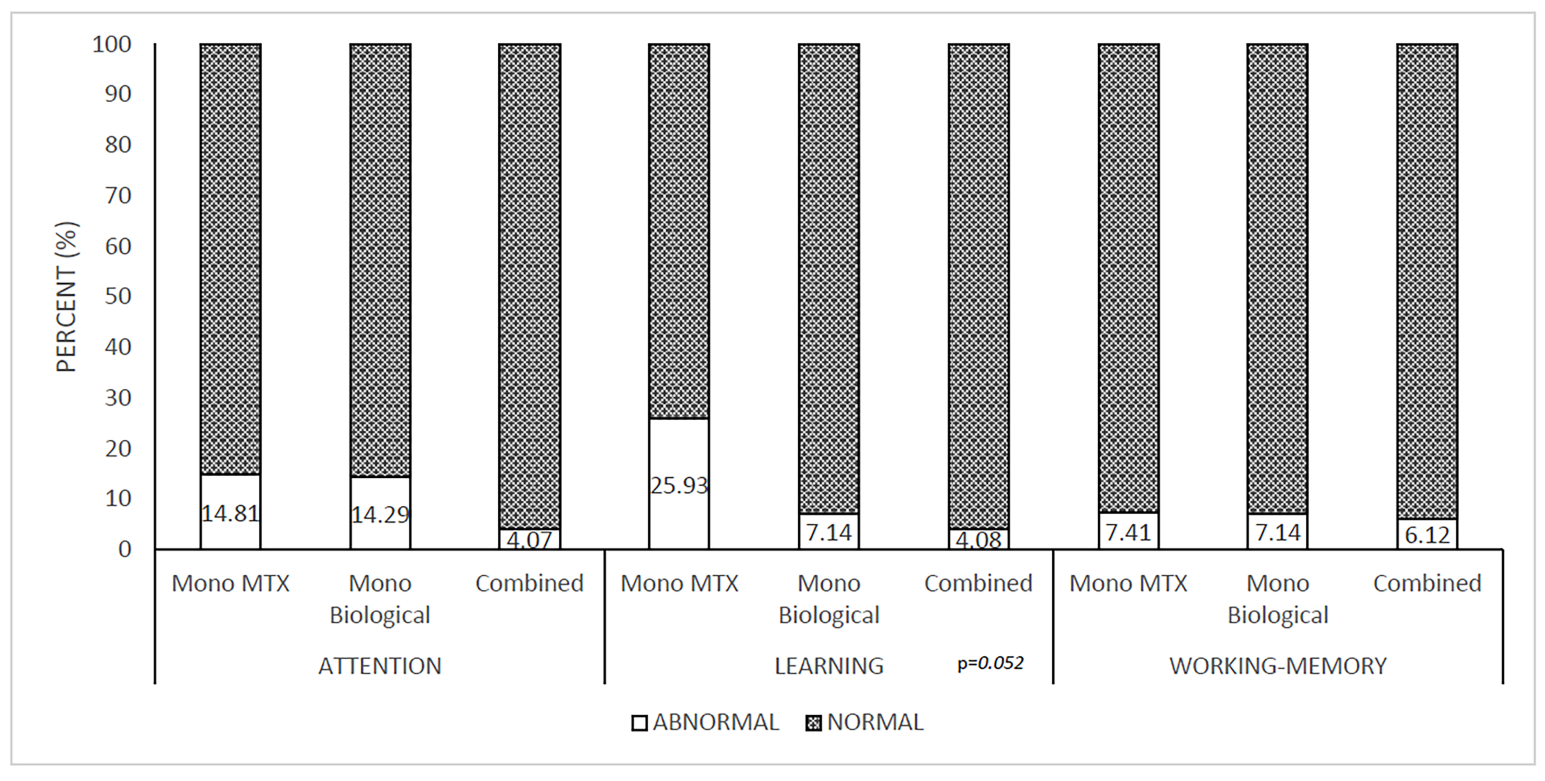
The distribution of normal and pathological neuropsychological parameters in different treatment groups. Mono MTX: methotrexate monotherapy; Mono Biological: TNF inhibitor monotherapy; Combined: (TNF inhibitor + methotrexate and/or sulfasalazine).

**Figure 2:**
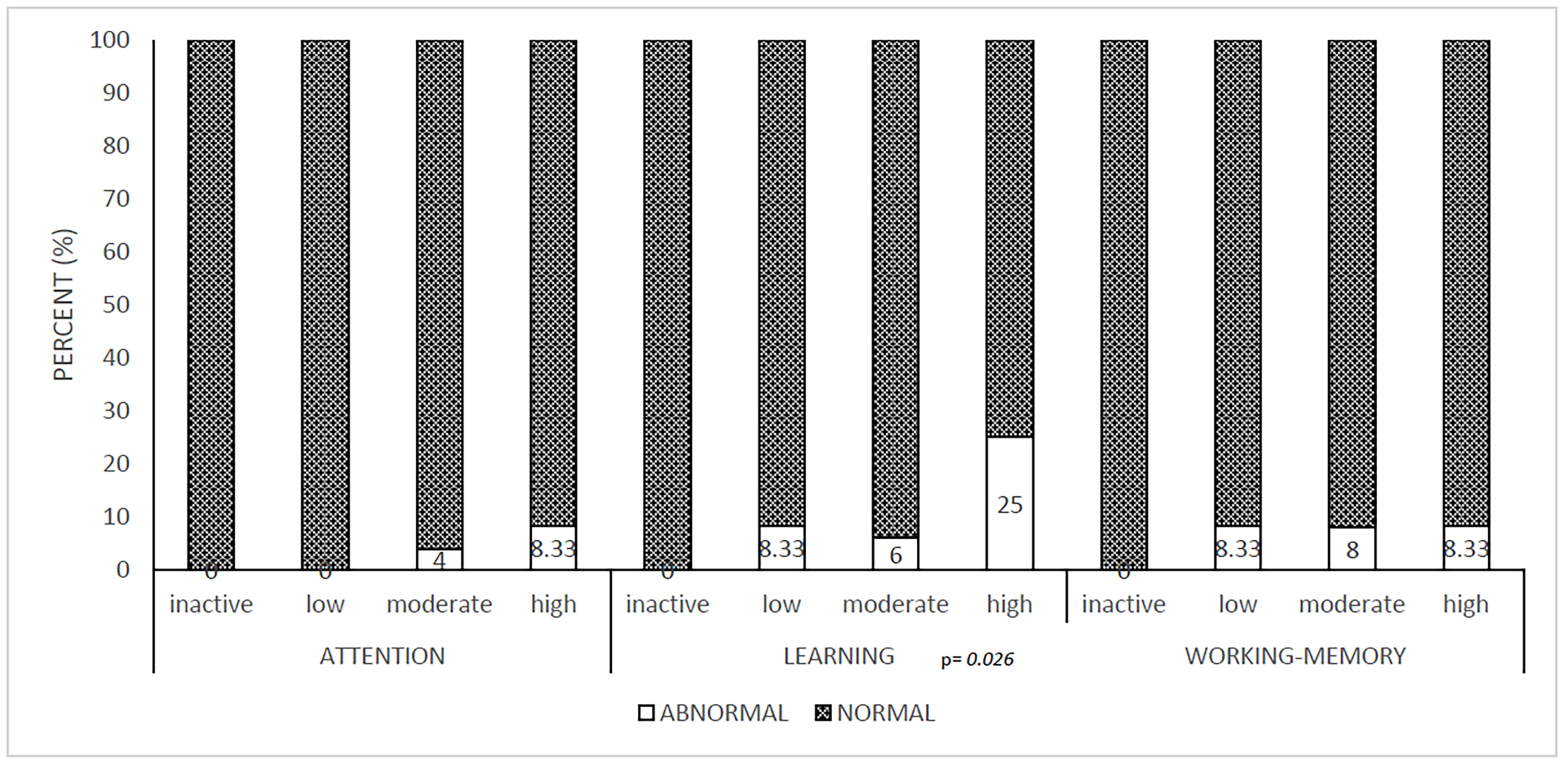
The distribution of normal and pathological neuropsychological parameters in different JADAS-71-I disease activity groups. JADAS-71-I: Juvenile Arthritis Disease Activity Score-71 before start of therapy.

Abnormally low values (≤89) showed similar rates across therapeutic and age groups, regardless of duration of therapy, following a normal distribution (Table 3). The MTX group showed a slightly higher incidence of abnormally low values, primarily in learning skills (Figure 1); however, no statistically significant differences were observed. Patients with high initial disease activity demonstrated significantly higher proportions of abnormally low learning skills compared to those with lower disease activity (Figure 2).

### 4.3. Treatment Group-Specific Neuropsychological Characteristics

a. Combination therapy group (n = 49): Recent disease activity significantly impacted working memory less in patients aged ≥11 years compared to younger subjects (*p* = 0.0288 for the interaction of age × JADAS-71-P). Average working memory remained within the normal range, suggesting limited clinical relevance.

b. MTX group (n = 27): Males exhibited significantly poorer learning skills, which were most pronounced in those with the highest baseline disease activity (gender: *p* = 0.0401; JADAS-71-I: *p* = 0.0312).

c. TNFi group (n = 14): The clinical relevance could not be determined due to the small sample size.

### 4.4. Factors That Affect Neuropsychological Scores

The type of therapy, gender, age at diagnosis, age at neuropsychological assessment, JADAS-71-P, and JADAS-71-I showed no impact on neuropsychological score trajectories.

### 4.5. Behavioral Skills Analysis

**Figure 3:**
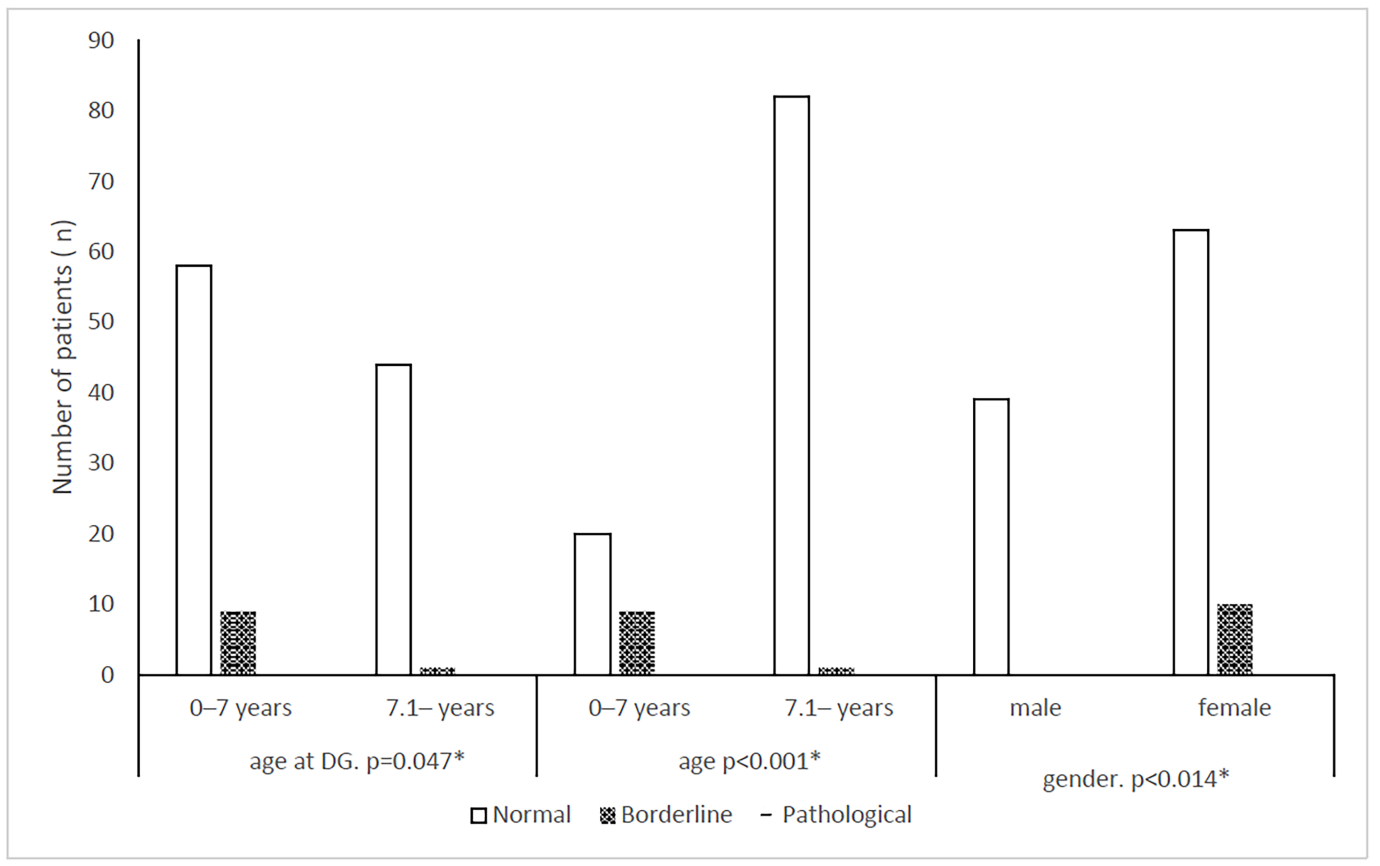
The distribution of patients with different scores of anxiety and depression by age and gender. Normal: number of patients with a normal range (0–90% rank) of values; borderline: number of patients with a borderline range (90.1–97% rank) of values; pathological: number of patients with an abnormal range (≥97.1% 1 rank) of values; age at DG: age at the date of diagnosis; age: age at the date of psychological examination, *: *p*-value is clinically significant.

Normal results were achieved in at least 83% (TNFi), 88% (MTX), and 90% (combination therapy) of patients. Pathological scores did not occur in the MTX group. Pathological values were observed in six patients for any behavioral skills. (There was a patient, who had more than one pathological parameters: aggression, deviant behavior, thinking, attachment, somatization, sociability scores). All were female, aged <7 years, with a history of disease onset before age 5, currently receiving combined therapy, five RF-negative polyarthritis, and one psoriatic arthritis. Pathological and borderline values were observed mainly in the patients diagnosed before age 7, with statistical significance in patients <7 years at psychological examination (Table 4). Patients with borderline/pathological scores had a mean age below 5 years at assessment (below 4 years at diagnosis). Gender evaluation showed significant difference for anxiety–depression, with borderline values significantly higher among females. No borderline or pathological values were detected in males. (Table 4, Figure 3). No differences were found in the distribution of normal, borderline, and pathological values between different therapeutic groups and disease activity levels.

It should be noted that the findings are observational and not causal due to the cross-sectional study design.

## 5. Discussion

We evaluated the neuropsychological status and behavioral characteristics of 112 patients with JIA, aged 2–18 years. This investigation was motivated by clinical observations and reports suggesting children with chronic arthritis experience more psychological problems than matched controls [4,24], though several previous publications reported contrasting findings [15,25,26]. Comparing studies from different time periods and countries with diverse cultural and economic backgrounds, therapeutic options, age groups, questionnaires, and sample sizes is not advisable without longitudinal follow-up. These differences contribute to the lack of expert consensus regarding associations between JIA and psychological problems [4,15,17].

We surveyed our JIA population to explore potential associations between disease-specific and demographic factors and behavioral or neuropsychological status, then compared psychological skills across treatment groups.

### 5.1. Neuropsychological Assessment

No significant between-group differences or associations with demographic and clinical parameters were observed for attention, learning, or working memory. Males in the MTX group demonstrated significantly poorer learning skills, which were most pronounced in those with the highest baseline disease activity. Although trends suggested lower learning skills in males, this did not emerge as a significant main effect in interaction studies with other variables. Therefore, we cannot conclude that males with JIA generally have lower learning ability scores. Overall, neuropsychological findings are reassuring, with no major pathological deviations observed during any therapy. Given expected results for attention, learning, and working memory, further investigation in different JIA subgroups was deemed unnecessary. We found no comparable study using our methodology in the literature. Similarly, reassuring results from a study reported cognitive profiles and estimated academic ability in JIA patients comparable to controls [27].

However, cognitive skill evaluations in adult JIA patients are less encouraging, showing lower cognitive function scores than healthy controls, particularly in visuospatial function [5]. Furthermore, a prospective follow-up study detected cognitive decline in almost one-third of adults with JIA [6]. Based on this information, we initiated re-evaluation of our own patients in 2025 to compare initial results with current data. In parallel, we have initiated a prospective longitudinal follow-up study with healthy controls.

### 5.2. Behavioral Skills

At least 83% of patients achieved normal results. Six patients (0–0.8% incidence) showed pathological results, which are considered rare occurrences. Psychological interventions were initiated for these patients. An Iranian study using the CBCL reported fewer positive results, with approximately 70% of JIA patients reaching borderline or clinically abnormal ranges in internalizing problems [4]—findings not replicated in our study. Pathological (aggression, deviant behavior, thinking, connection, somatization, and sociability) and borderline values occurred only in patients diagnosed before age 7. Methodological differences limit direct comparison between studies.

Studies on mood disorders in adults with JIA yield mixed results. Packham et al.’s review found several pre-2004 studies showing higher anxiety and depression risk in JIA patients, possibly due to the limited effectiveness of therapies at that time [18]. However, 2024 studies on mood disorders with current, more effective therapies found similarly concerning results [7,28]. Previous research demonstrated that age at disease onset and gender significantly influence psychological outcomes in patients with JIA [27,29]. Younger children diagnosed with JIA show an increased likelihood of developing behavioral disorders, while adolescents—particularly females—demonstrate greater susceptibility to anxiety and depression. The etiology of these behavioral changes is complex (developmental vulnerability, gender-specific factors, early disease onset impact, parental perception biases, and coping mechanism development). A chronic illness that develops in early childhood may foster overprotective parental attitudes, impacting psychological development. Comparing behavioral skills between early-onset JIA and other early-onset chronic diseases could determine the disease specificity of these observations.

Previous studies inconsistently suggest patients with JIA have normal internalizing and externalizing behaviors [17,29,30]. Our results support findings that anxiety/depression onset in adults with JIA correlates with age at diagnosis, with mood disorders more pronounced among those diagnosed between ages 6 and 12.6 [31]. In our cohort, all patients in the pathological and borderline ranges were <7 years old (<5 at diagnosis). While parental personality may influence questionnaire completion and potentially bias results, self-report forms are unavailable for children < 7 years. Therefore, combined psychological examination and parental questionnaires were evaluated together to reduce interpretation bias.

We recommend closer psychological monitoring for patients diagnosed with JIA before age 5, with timely psychologist involvement. Age at JIA onset may influence neuropsychological development and adult psychological status.

Similarly to previous studies, we observed female predominance in anxiety–depression susceptibility. Although no patients showed abnormal anxiety/depression scores, borderline values occurred. These data underscore the need for age- and gender-specific psychological support and interventions.

Disease activity did not affect behavioral skills, consistent with Ding et al., who found no negative impact of disease activity or disability on psychological well-being in polyarticular JIA [17]. However, disability presence further limits community participation, fostering the development of isolation and depression and correlating with disability degree [19]. We could not assess the impact of disability on anxiety, as patients’ CHAQ and JADI scores indicated minimal disability. Roemer and Milatz recently published risk factors for depressive symptoms in JIA [7,28]. Unlike our results, they found a significant association between depressive symptoms and increased disease activity. Both examined larger cohorts using different assessment tools; their thought-provoking results warrant continued research and systematic psychological follow-up.

Among patients with pathological behavioral skills, we detected five RF-negative polyarthritis cases and one psoriatic arthritis case. Patients with enthesitis-related arthritis (ERA) showed no internalized problem or anxiety/depression despite data from the literature on their intensity of pain [32,33]. In a recent study, different JIA subtypes were examined: spondyloarthropathies and polyarticular JIA patients had worse total competence and internalizing scores than healthy controls. Self-harm/suicide rates were almost four times higher in patients with polyarticular JIA than healthy controls [34]. Despite similar patient numbers (111 vs. 112), we found no suicidal tendencies. Our study design precluded analysis of the effects of disease burden on different JIA subtypes due to the under-representation of some subtypes.

### 5.3. Our Study Has Limitations

Our observational findings cannot establish causality due to the cross-sectional design. Interpretation of the results must consider that the study did not encompass all factors influencing child psychological development, with measurements conducted only once at a single center. We lack healthy, matched controls for comparison, although abnormal scores in our questionnaire represent deviations from normal, healthy populations. Parents completed questionnaires; to reduce bias, each child underwent a detailed psychological evaluation, with medical history recorded before questionnaire completion. We did not separately investigate the overprotective attitude of family, an important factor with respect to cognitive abilities [18]. Parenting styles, mental health awareness, and societal expectations regarding child development have evolved since the original data collection, potentially affecting parental perception and reporting of child behavior, particularly in questionnaire-based instruments like the CBCL. Although the overall sample size is satisfactory, some subgroup or stratum comparisons were not possible due to small sample sizes.

## 6. Conclusions

Neuropsychological performance of patients with JIA was not adversely affected by demographic, disease-related, or treatment factors. Pathological behavioral results were rare in our cohort. Females with early-onset JIA warrant close monitoring throughout the disease course. Psychological screening and follow-up remain crucial for achieving long-term improvements in quality of life. Understanding whether JIA and various therapies affect the psychological well-being of children requires future research, including extensive follow-up studies using standardized tools, outcome measures, and clearly classified patient groups.

## Figures and Tables

**Table 1:** Characteristics and neuropsychological results of patients (N = 90) in different treatment groups.

	MTX (n = 27)	TNF Inhibitor (n = 14)	Combination (n = 49)	*p*-Value
Female, n (%)	17 (62.9)	11 (78.6)	27 (55.1)	0.275 ^a^
Age at diagnosis, years	8.17 ± 4.68	6.22 ± 4.55	6.32 ± 4.57	0.154 ^b^
Age at psychological assessment, years	10.45 ± 4.03	10.41 ± 4.36	10.45 ± 4.34	0.686 ^b^
Duration of therapy, years	1.56 ± 1.44	1.98 ± 1.39	1.92 ± 1.36	0.056 ^b^
JADAS-71 I score	5.94 ± 3.10	9.34 ± 3.99	9.28 ± 3.99	0.0065 ^b^ (MTX vs. Combined: 95% CI (0.80, 5, 17 ***)
JADAS-71 P score	2.99 ± 2.92	2.88 ± 2.57	2.84 ± 2.49	0.165 ^b^
JIA subtype, n (%)				NA
Oligo	6 (22.22)	0 (0)	2 (4.08)	
Extended oligo	2 (7.4)	5 (35.71)	12 (24.50)	
RF+ poly	1 (3.71)	0 (0)	2 (4.08)	
RF− poly	4 (14.81)	3 (21.42)	23 (46.94)	
ERA	6 (22.22)	4 (28.57)	3 (6.12)	
PsA	1 (3.71)	1 (7.14)	1 (2.04)	
UA	7 (25.93)	1 (7.14)	6 (12.24)	
Attention (score, normal range: 90–110)	108.78 ± 19.30	108.64 ± 18.45	111.90 ± 17.58	0.715 ^b^
Learning (score, normal range: 90–110)	99.96 ± 14.05	107.29 ± 14.97	105.90 ± 12.92	0.134 ^b^
Working memory (score, normal range: 90–110)	110.85 ± 14.35	110.14 ± 17.73	110.29 ± 12.71	0.982 ^b^

(Values are mean ± SD or n (%). JIA: juvenile idiopathic arthritis; Oligo: oligoarticular JIA; RF+ poly: rheumatoid factor-positive JIA; RF− poly: rheumatoid factor-negative JIA; ERA: enthesitis-related arthritis; PsA: psoriatic arthritis; UA: undifferentiated arthritis; NA: not applicable; ^a^: chi-square test; ^b^: one-way ANOVA test. *** Selected by Tukey’s post hoc test (CI) for the mean difference.

**Table 2:** Characteristics of patients (n = 112) and distribution of behavioral variables—CBCL questionnaire results.

	MTX (n = 34)	TNF Inhibitor (n = 18)	Combination (n = 60)	*p*-Value
Patient characteristics				
Female, n (%)	21 (83.33)	15 (61.76)	37 (61.67)	0.210 ^a^
Age at diagnosis, years	7.89 ± 4.74	6.22 ± 4.55	6.26 ± 4.57	0.797 ^b^
Age at psychological assessment, years	10.11 ± 4.21	10.41 ± 4.36	10.36 ± 4.39	0.732 ^b^
Duration of therapy, years	1.54 ± 1.41	1.98 ± 1.39	1.91 ± 1.36	0.052 ^b^
JADAS-71 I, score	5.82 ± 3.08	9.34 ± 3.99	9.28 ± 3.96	0.0001 ^b^ (MTX vs. Combined: 95% CI (1.68, 5.52) MTX vs.TNF inhibitor: 95% CI (0.21, 5.41) ^c^
JADAS-71 P, score	2.89 ± 2.88	2.88 ± 2.57	2.83 ± 2.47	0.118 ^b^
Behavioral variables (N_normal_/N_borderline_/N_pathological_)				
Attachment	31/3/0	16/2/0	55/4/1	0.840 ^a^
Somatization	31/3/0	16/2/0	56/3/1	0.795 ^a^
Anxiety/depression	31/3/0	17/1/0	54/6/0	1.000 ^a^
Sociability	30/4/0	16/2/0	56/3/1	0.599 ^a^
Thinking	31/3/0	16/1/1	55/5/0	0.464 ^a^
Deviant behavior	30/4/0	16/2/0	56/3/1	0.599 ^a^
Attention	30/4/0	15/3/0	57/3/0	0.152 ^a^
Aggression	30/4/0	17/1/0	54/5/1	0.901 ^a^

Values are mean ± SD or n (%); ^a^: chi-square test/Fisher’s exact test; ^b^: one-way ANOVA test; ^c^: selected by Tukey’s post hoc test (CI) for the mean difference.

**Table 3:** The distribution of normal and pathological values of neuropsychological parameters.

	Attention (N_normal_/N_pathological_)	Learning (N_normal_/N_pathological_)	Working Memory (N_normal_/N_pathological_)
Therapy type			
Combined	47/2	46/3	46/3
MTX	23/4	20/7	25/2
TNFi	12/2	13/1	13/1
*p*-value	0.159	0.052	1.000
Age at diagnosis			
0–7 years	41/4	41/4	42/3
7.1 years	41/4	38/7	42/3
*p*-value	1.000	0.521	1.000
Age at NP assessment			
6–11 years	41/2	38/5	42/1
11.1 years	41/6	41/6	42/5
*p*-value	0.270	1.000	0.205
Duration of therapy			
0–2 years	53/6	51/8	55/4
2.1 years	29/2	28/3	29/2
*p*-value	0.709	0.742	1.000
Gender			
male	32/3	29/6	33/2
female	50/5	50/5	51/4
*p*-value	1.000	0.326	1.000
JADAS-71-I			
low	12/0	11/1	11/1
moderate	40/5	43/2	42/3
high	30/3	25/8	31/2
*p*-value	0.684	0.026 *	1.000
JADAS-71-P			
inactive	23/2	23/2	24/1
low	34/3	34/3	34/3
moderate	17/2	15/4	18/1
high	8/1	7/2	8/1
*p*-value	1.000	0.318	0.819

N normal/N pathological: number of patients with normal values (90 and above)/number of patients with abnormally low values (<90); TNFi: TNF inhibitor; NP: neuropsychological; JADAS-71-I: JADAS-71 disease activity before start of therapy; JADAS-71-P: JADAS-71 disease activity at the time of psychological assessment, *: *p*-value is clinically significant.

**Table 4:** The distribution of behavioral variables in different subgroups.

(N_n_/N_b_/N_p_)	Attachment	Somatization	Anxiety–Depression	Sociability	Thinking	Deviant Behavior	Concentration	Aggression
Age at diagnosis								
0–7 years	57/9/1	59/7/1	58/9/0	57/9/1	57/9/1	57/9/1	57/10/0	56/10/1
≥7.1. years	45/0/0	44/1/0	44/1/0	45/0/0	45/0/0	45/0/0	45/0/0	45/0/0
*p*-value	0.010 *	0.188	0.047 *	0.010 *	0.010 *	0.010 *	0.005 *	0.005 *
Age at psychological assessment								
0–7 years	20/9/0	21/7/1	20/9/0	19/9/1	19/9/1	19/9/1	20/9/0	18/10/1
≥7.1.years	82/1/0	82/1/0	82/1/0	83/0/0	83/0/0	83/0/0	82/1/0	83/0/0
*p*-value	<0.001 *	<0.001 *	<0.001 *	<0.001 *	<0.001 *	<0.001 *	<0.001 *	<0.001 *
Duration of therapy								
0–2 years	69/9/1	71/8/0	70/9/0	70/8/1	70/8/1	70/8/1	69/10/0	69/9/1
2.1– years	33/0/0	32/1/0	32/1/0	32/1/0	32/1/0	32/1/0	33/0/0	32/1/0
*p*-value	0.073	0.036 *	0.276	0.489	0.489	0.489	0.032 *	0.489
Gender								
Male	36/3/0	37/2/0	39/0/0	37/2/0	*38/1/0*	38/1/0	38/1/0	37/2/0
Female	66/6/1	66/6/1	63/10/0	65/7/1	*64/8/1*	64/8/1	64/9/0	64/8/1
*p*-value	1.000	0.811	0.014 *	0.667	0.195	0.195	0.160	0.667
JADAS-71-I								
Low	12/0/0	12/0/0	12/0/0	12/0/0	12/0/0	12/0/0	12/0/0	12/0/0
Moderate	51/5/0	52/4/0	49/7/0	50/5/1	50/5/1	47/8/1	51/5/0	49/6/1
High	39/4/1	39/4/1	41/3/0	40/4/0	40/4/0	43/1/0	39/5/0	40/4/0
*p*-value	0.719	0.650	0.446	0.890	0.890	0.090	0.636	0.814
JADAS-71-P								
Inactive	31/1/0	28/4/0	29/3/0	28/3/1	28/4/0	28/4/0	27/5/0	29/3/0
Low	41/6/1	44/3/1	44/4/0	44/4/0	43/4/1	43/4/1	44/4/0	43/5/0
Moderate	21/1/0	22/0/0	19/3/0	21/1/0	21/1/0	21/1/0	21/1/0	20/1/1
High	9/1/0	9/1/0	10/0/0	9/1/0	10/0/0	10/0/0	10/0/0	9/1/0
*p*-value	0.564	0.498	0.802	0.831	0.889	0.889	0.498	0.692

Nn/Nb/Np: number of patients with a normal range (0–90% rank) of values/number of patients with a borderline range (90.1–97% rank) of values/number of patients with pathological = abnormal range (≥97.1% rank) of values; JADAS-71-I: JADAS-71 disease activity before start of therapy; JADAS-71-P: JADAS-71 disease activity at the time of psychological assessment, *: *p*-value is clinically significant.

## Data Availability

The datasets used and/or analyzed during the current study are available from the corresponding author upon reasonable request.

## Abbreviations

JIA: juvenile idiopathic arthritis; SJIA: systemic-onset juvenile idiopathic arthritis; ERA: enthesitis-related arthritis; Poly JIA: polyarticular juvenile idiopathic arthritis; RF: rheumatoid factor; MTX: methotrexate; SSZ: sulfasalazine; CBCL: Child Behavior Checklist; AE: adverse event; NP: Neuropsychological; JADAS-71: Juvenile Arthritis Disease Activity Score-71; JADAS-71-I: Initial JADAS-71 (before treatment); JADAS-71-P: JADAS-71 at psychological assessment.
